# Supplementary material for: Atomic structure evolution related to the Invar effect in Fe-based bulk metallic glasses
Source: Nat Commun. 2022 Feb 28;13:1082. doi: 10.1038/s41467-022-28650-9 (PMC8885758; doi:10.1038/s41467-022-28650-9)
Supplement: Supplementary file 1 — Supplementary Information [file 41467_2022_28650_MOESM1_ESM.pdf]

Supplementary Information for "Atomic structure evolution related to the Invar effect in Fe-based bulk metallic glasses"

Alexander Firlus[1]*, Mihai Stoica[1], Stefan Michalik[2], Robin E. Schäublin[1], Jörg F. Löffler[1]*

Affiliations

[1] Laboratory of Metal Physics and Technology, Department of Materials, ETH Zurich, 8093 Zurich, Switzerland

[2] Diamond Light Source Ltd., Harwell Science and Innovation Campus, Didcot, Oxfordshire, OX11 0DE, UK

Corresponding authors

Correspondence to:

Alexander Firlus (alexander.firlus@mat.ethz.ch),

Jörg F. Löffler (joerg.loeffler@mat.ethz.ch)

Supplementary material

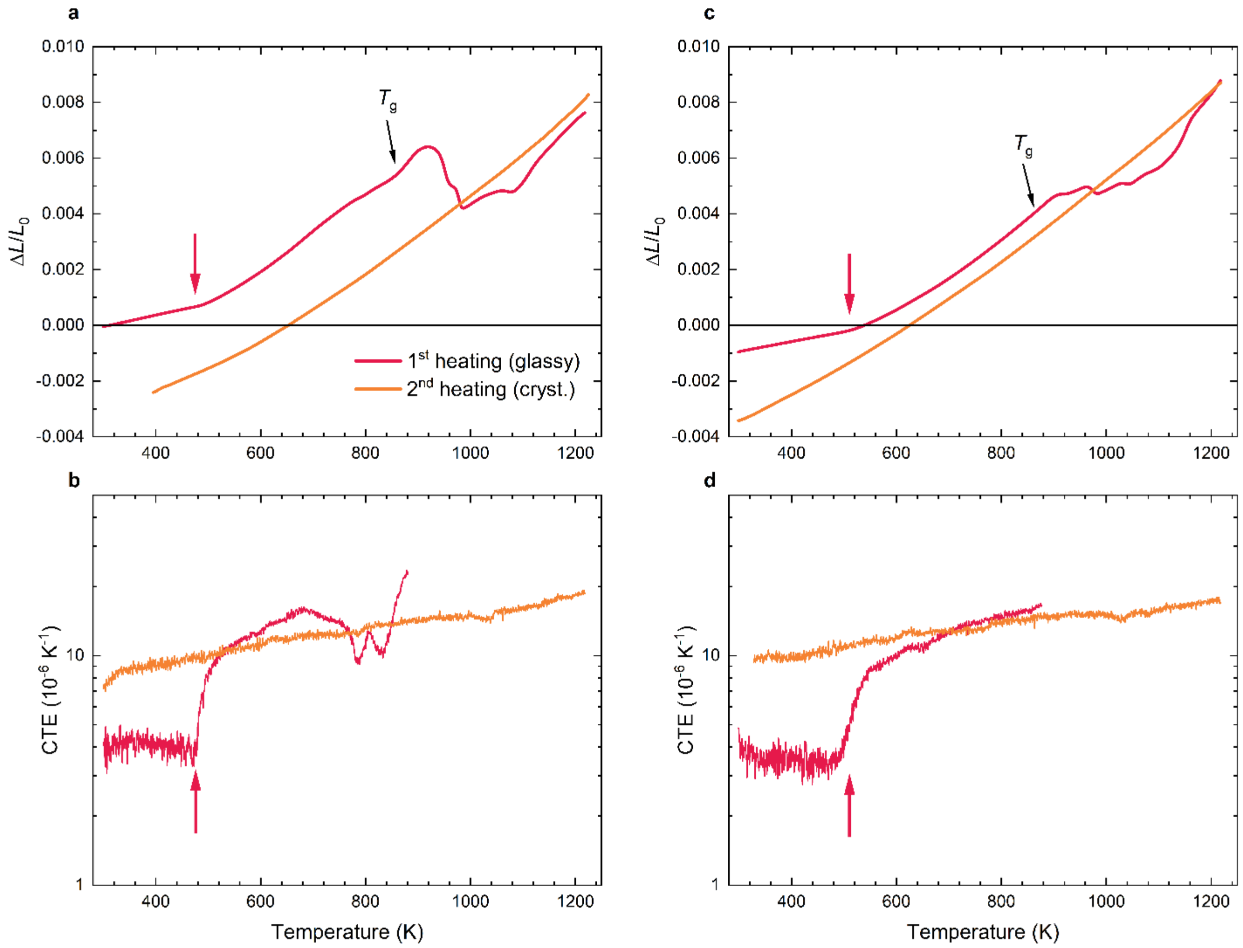

Supplementary Figure 1. Dilatometry on QNb and QMo. The dilatometric transition temperature is marked with a red arrow. (a) The length change of QNb reveals the dilatometric transition temperature and glass transition temperature, with no further transitions occurring after the sample has crystallized. (b) The CTE of QNb increases by a factor of 3 at the dilatometric transition temperature, whereas the fully crystallized sample shows no Invar effect anymore. (c) The length change of QMo also shows the Invar effect only in the glassy state. (d) The CTE of QMo increases by a factor of 4 at the dilatometric transition temperature for the glassy sample, whereas the Invar effect disappears once the sample has crystallized.

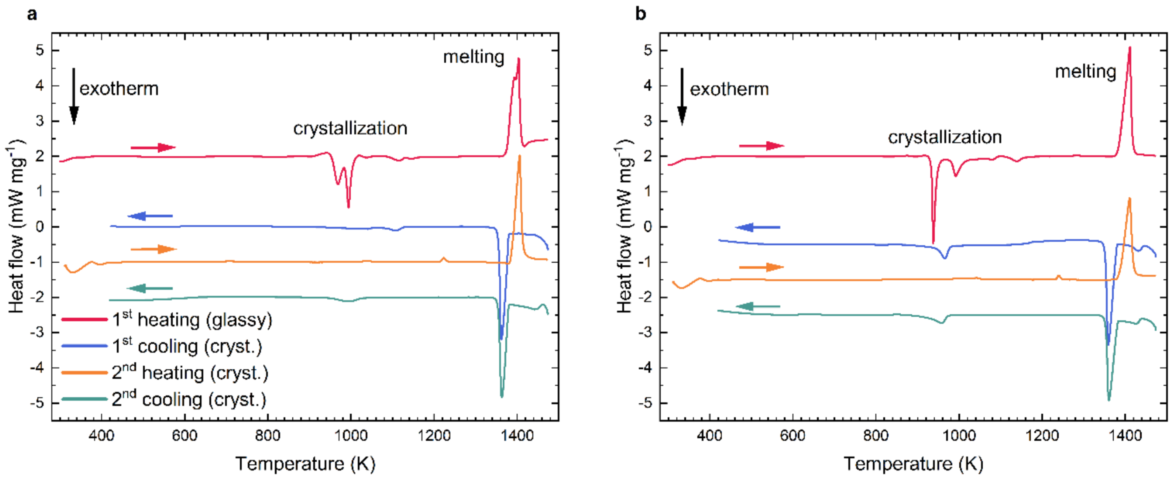

Supplementary Figure 2. DSC curves of (a) QNb and (b) QMo. All curves were measured at a rate of 20 K/min. For better clarity they are vertically offset. The crystallization events begin at 951 K and 923 K for QNb and QMo, respectively. The DSC curves are in agreement with previous studies of these alloys[5,14,15].

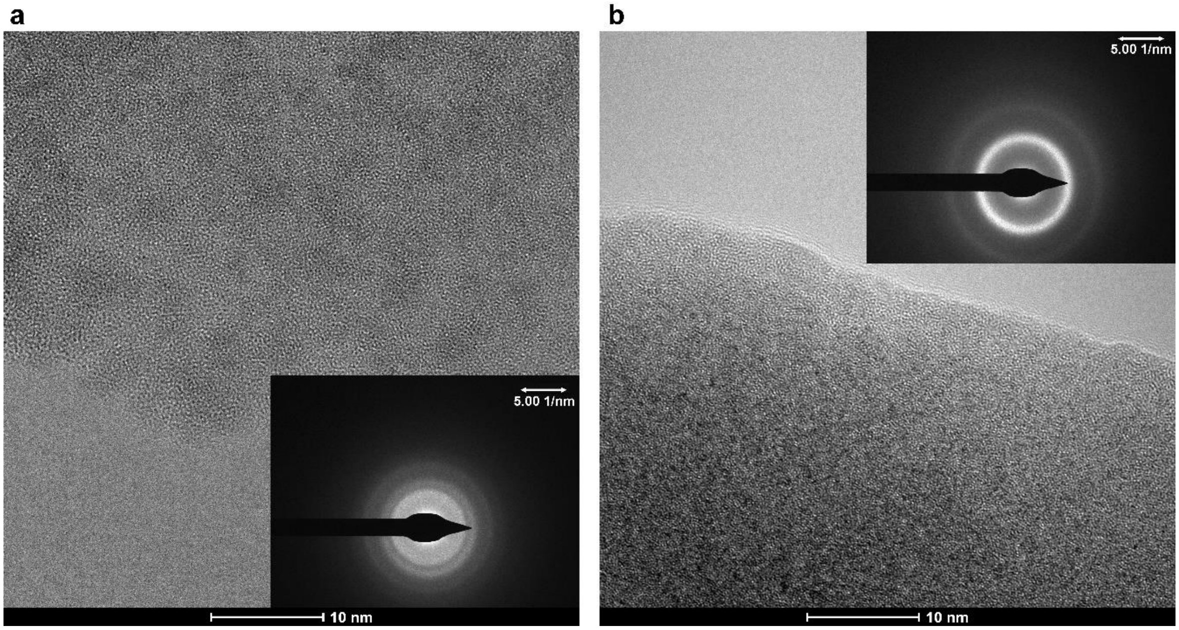

Supplementary Figure 3. High-resolution transmission electron microscopy of (a) QNb and (b) QMo. Both samples are completely amorphous. The insets show the selected area diffraction patterns.

Supplementary Table 1. Chemical composition of QNb and QMo measured by inductively coupled plasma optical emission spectroscopy (ICP-OES).

	Fe	B	Y	Nb	Mo	Gd/Ta
QNb [wt.%]	78.21	6.03	7.94	7.82	< 0.004	< 0.01
QMo [wt.%]	77.67	4.55	8.08	< 0.01	9.69	< 0.01
